# How Effective Is Pulse Arrival Time for Evaluating Blood Pressure? Challenges and Recommendations from a Study Using the MIMIC Database

**DOI:** 10.3390/jcm8030337

**Published:** 2019-03-11

**Authors:** Yongbo Liang, Derek Abbott, Newton Howard, Kenneth Lim, Rabab Ward, Mohamed Elgendi

**Affiliations:** 1 School of Electrical and Computer Engineering, University of British Columbia, Vancouver BC V6T 1Z4, Canada; liangyongbo001@gmail.com (Y.L.); rababw@ece.ubc.ca (R.W.); 2 School of Electrical and Electronic Engineering, The University of Adelaide, Adelaide SA 5005, Australia; derek.abbott@adelaide.edu.au; 3 Centre for Biomedical Engineering, The University of Adelaide, Adelaide SA 5005, Australia; 4 Nuffield Department of Surgical Sciences, University of Oxford, Oxford OX3 9DU, UK; newton.howard@nds.ox.ac.uk; 5 Howard Brain Sciences Foundation, Providence, RI 02906, USA; 6 Faculty of Medicine, University of British Columbia, Vancouver BC V1Y 1T3, Canada; klim@cw.bc.ca; 7 BC Children’s & Women’s Hospital, Vancouver BC V6H 3N1, Canada

**Keywords:** pulse morphology, pulse oximeter, blood pressure monitoring, pulse arrival time, global health, digital medicine, wearable devices, hypertension assessment, hypertension evaluation

## Abstract

Cardiovascular disease (CVD) is the number one cause of non-infectious morbidity and mortality in the world. The detection, measurement, and management of high blood pressure play an essential role in the prevention and control of CVDs. However, owing to the limitations and discomfort of traditional blood pressure (BP) detection techniques, many new cuff-less blood pressure approaches have been proposed and explored. Most of these involve arterial wave propagation theory, which is based on pulse arrival time (PAT), the time interval needed for a pulse wave to travel from the heart to some distal place on the body, such as the finger or earlobe. For this study, the Medical Information Mart for Intensive Care (MIMIC) database was used as a benchmark for PAT analysis. Many researchers who use the MIMIC database make the erroneous assumption that all the signals are synchronized. Therefore, we decided to investigate the calculation of PAT intervals in the MIMIC database and check its usefulness for evaluating BP. Our findings have important implications for the future use of the MIMIC database, especially for BP evaluation.

## 1. Introduction

Cardiovascular disease (CVD) is the largest non-infectious cause of morbidity and mortality across the globe. The prevention and management of CVD have been challenging for a number of reasons including the detection and management of excessively high blood pressure (hypertension). The most widely utilized clinical method of measuring blood pressure is done via a sphygmomanometer consisting of an inflatable cuff attached to the measurement device. Although it is the clinical gold standard, this method of measurement has challenges, such as limited convenience and mobility, the need for frequent measurements, and variability in fitting the cuff. Alternative methods of assessing blood pressure (BP) to overcome those challenges have gained traction in recent years.

In recent years, the arterial wave propagation theory, which relies on simultaneously collected electrocardiogram (ECG) and photoplethysmogram (PPG) signals for BP detection, has attracted the interest of many researchers [1]. This theory is based on the idea of wave propagation on a certain path. The start point and end point signals of the path are extracted, and the pulse transit time (PTT), pulse arrival time (PAT), and pulse wave velocity (PWV) can be calculated and used to determine cardiovascular function statuses, such as blood pressure, arterial stiffness [2], and arterial compliance. Electrocardiograms (ECG), impedance cardiographs (ICG), ballistocardiographs (BCG), and phonocardiograms (PCG) are the most common ways to determine or measure the start point of the PAT [3,4], and photoplethysmography (PPG) from the finger is the most common way to determine the end of the PAT. The PAT-based approach for cuff-less BP detection is being used regularly in current studies.

To our knowledge, the Medical Information Mart for Intensive Care (MIMIC) database [5] is the only publicly available database in which ECG, PPG, and arterial blood pressure (ABP) signals were simultaneously collected (i.e., all signals were collected at the same time with no delay). Many papers have been published with results based on the MIMIC database and on the assumption that all the signals were collected at the same time and synchronized [6,7,8,9,10]. A recent paper [11] discussed a feature that relies on the synchronicity between PPG and ECG, using the MIMIC database, and also discussed features that do not rely on synchronicity, extracted from PPG waveforms. However, the researchers who created the database have stated that there are errors in the data matching and alignment, indicating that some (perhaps all) recordings were not synchronized [12]. This contradiction motivated us to investigate the MIMIC database to report challenges and provide some recommendations for better use of the database in BP evaluation.

This study focuses on examining the correlation of PAT and BP using the MIMIC database. PAT is the main parameter being investigated and the MIMIC database is the most used public database for evaluating blood pressure. The MIMIC database is an influential database, and it is anticipated that an increased amount of blood pressure prediction research will be conducted based on ECG and PPG signals. Study results will be valuable and meaningful and will help many researchers to utilize the database effectively. Moreover, we assessed the quality of the ECG, PPG, and ABP signals, and recommended the records of high quality for future use and that records of low quality need to be excluded in future use. The rigor evaluation of PAT and the signal quality may be useful for researchers who are willing to investigate the BP using the MIMIC database.

## 2. Experimental Section

### 2.1. Data Collection

The MIMIC database [5,13] was used as the data source for this study. For our purposes, record collection was confined to records that contained physiological information only, such as gender and age, and included waveforms with ABP, ECG, and PPG signals. The MIMIC database is created from data collected from intensive care units (ICUs), and there are many factors that could corrupt the collected signals; therefore, it is difficult to ensure the high quality of ABP, ECG, and PPG signals [14]. It was essential to obtain acceptable records; accordingly, a total of 578 subjects’ original records were downloaded through Physionet ATM, which has the longest records for each subject (8000 s, 2.2 h). However, as mentioned above, many records were inadequate and included, for example, one or two missing signals and abnormal (low quality) signals.

**Figure 1:**
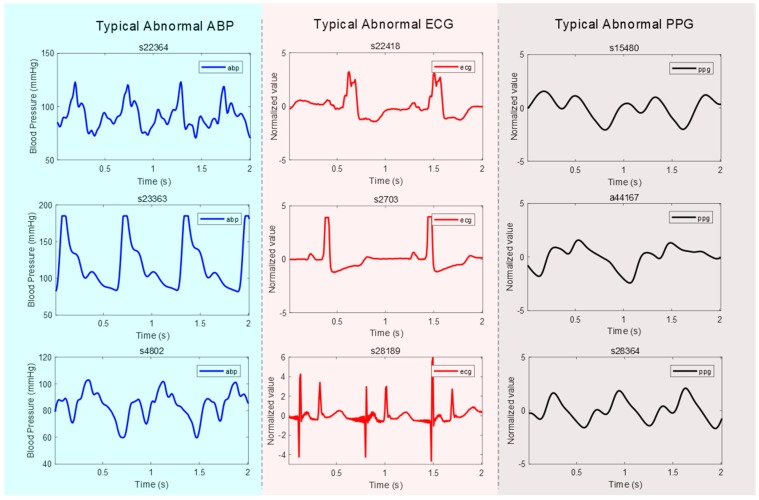
Annotations and typical cases of abnormal waveforms that were excluded from this study. Note that ABP is the arterial blood pressure, ECG is the electrocardiogram, and PPG is the photoplethysmogram signal.

Excluding subjects with missing PPG or ECG signals is a straightforward process. However, for excluding abnormal signals, we used the following rules for consistency:

1. **Abnormal ABP signal**: An “abnormal” ABP signal refers to an ABP signal where the systolic and diastolic waves cannot be distinguished, or their morphologies are highly distorted, as shown in Figure 1;
2. **Abnormal ECG signal**: An “abnormal” ECG signal refers to an ECG signal where the morphology of the QRS waves is highly distorted, as shown in Figure 1;
3. **Abnormal PPG signal**: An “abnormal” PPG signal refers to a PPG signal where the systolic and diastolic waves cannot be distinguished, their morphologies are highly distorted, and heart rate cannot be determined, as shown in Figure 1.

To minimize signal interference in this study, some of these record types were annotated and excluded, for example, as “missing signal” or “abnormal signal.” Examples of these types are shown in Figure 1. The statistics of these types of records are summarized in Table 1. All included signals in the analysis are stated in Table 1.

**Figure 2:**
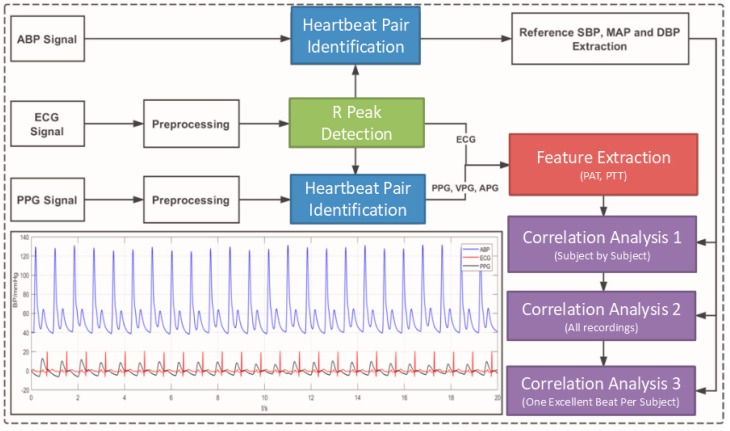
The workflow of this study. ABP is the arterial blood pressure, ECG is the electrocardiogram, PPG is the photoplethysmogram signal, VPG is the velocity of PPG, APG is the acceleration of PPG, PTT is the pulse transit time, and PAT is the pulse arrival time.

After inclusion and exclusion processing, 121 records from 121 subjects were archived in the dataset of this study. They each had a unique ID and the same waveform length (120 s, 125 Hz sample frequency). The subjects were 64 ± 17 years old. The workflow of this study is shown in Figure 2.

### 2.2. Signal Preprocessing and Feature Definition

Each waveform record in the dataset of this study had an ABP, ECG, and PPG signal. The ABP signal was used to extract systolic blood pressure (SBP) and diastolic blood pressure (DBP) as the reference blood pressure value. ECG and PPG signals were used to extract the PATs.

**Figure 3:**
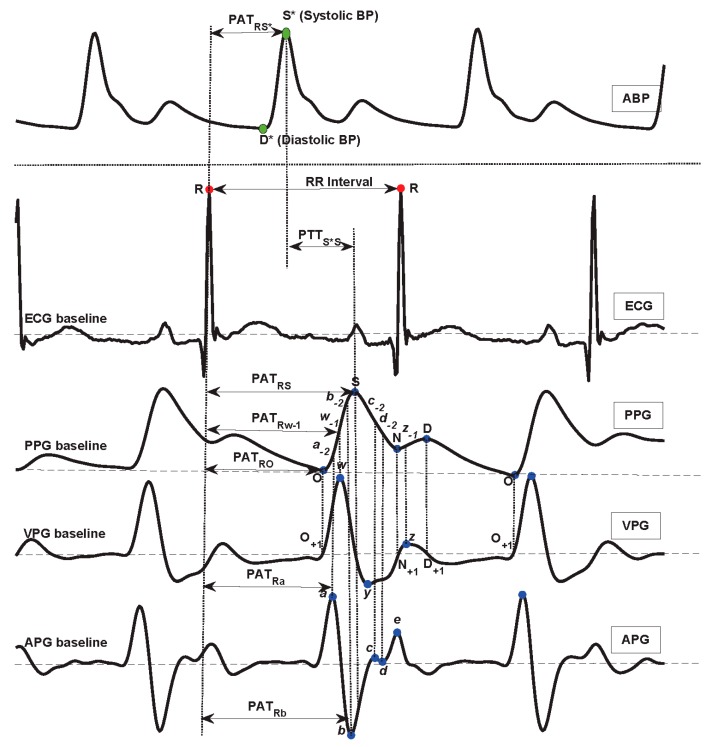
The characteristic point definition in ABP, ECG, PPG, VPG, and APG waveforms. Here, PTT is the duration between two arterial sites, PAT is the duration between the ECG R peak and a distal arterial waveform, ABP is the arterial blood pressure, ECG is the electrocardiogram, PPG is the photoplethysmogram signal, VPG is the velocity of PPG, APG is the acceleration of PPG, PTT is the pulse transit time, and PAT is the pulse arrival time.

It is worth mentioning that PAT and PTT are used interchangeably; however, Figure 3 shows the difference between the PAT and PTT durations. The PAT is defined as the period from the start point of the ECG heartbeat (usually the R peak is used as a label for a heartbeat) to a distal arterial waveform (usually the systolic peak of the PPG signal measured from the fingertip), while the PTT is defined as the period from the arterial site (usually the systolic peak of the ABP signal measured from the upper arm) to another arterial site (usually the systolic peak of the PPG signal measured from the fingertip) [1]. Note that the PAT duration includes the PTT duration. There is another duration that can be used with the PAT and PTT for refining BP estimation, called the pre-ejection period (PEP) [15]; however, this PEP period was not investigated in the present study, as the main concern was to examine the synchronicity between signals.

It is worth mentioning that PAT and PTT durations are usually measured from pulse foot to pulse foot. The foot of the pulse is determined by first finding the pulse peak, and then by searching for the beginning of the pulse. If the pulse peak is not clear for any reason (such as noise or abnormality), determining the foot becomes even more difficult. Therefore, researchers prefer to use peak to peak durations rather than foot to foot durations. Also, researchers have started to apply mathematical derivatives to the signal to emphasize the weak peaks. Applying the first derivative to the PPG signal provides a signal called the velocity photoplethysmogram (VPG), while applying the second derivative to the PPG signal provides the acceleration photoplethysmogram (APG).

The fiducial points used to define the PPG, VPG, and APG waveform are partly described in [16] and fully described in [17]. We followed the recommendations in [17] for naming the waves for consistency and for more effective reproducibility. This enhanced the benefits and potential of using PPG waveforms for investigating different diseases. Note that the acronyms PPG, VPG, and APG were used according to the recommendations in [18].

Interestingly, the fiducial points used to define the start and end points for the PTT and PAT intervals are also not consistent in the literature, but some general convention exists. While the R-wave of an ECG and the maximum slope point of a PPG signal are perhaps the most commonly used fiducial points, studies have shown that selecting different fiducial points in the PPG waveform differentially impacts the accuracy of BP calculations. In 2013, Choi et al. [19] tested three different measurement points for PAT, including the peak, middle, and end of the PPG waveform, which were defined as PAT-peak (PAT_RS_), PAT-middle (PAT_R*w-1*_), and PAT-foot (PAT_RO_), respectively, as shown in Figure 3. The PAT-middle is the maximum slope point. Their study showed that PAT has a strong correlation with SBP and DBP and that PAT-middle is the best measure to use in terms of minimizing standard error deviations. Cattivelli and Garudadri [20] and Li et al. [21] performed similar studies and found similar results. Generally, the PAT-middle makes more sense clinically, as does the R-peak in ECGs (representing the onset of ventricular mechanical contraction in the heart), while the middle of the PPG waveform (the peak of the first derivative of the PPG signal) is the moment when a pulse wave transmits to the artery. In this respect, PAT-middle is the time delay between the contraction phase beginning at the heart and the subsequent contraction of the artery [22].

Meanwhile, the PPG and its derivatives, which contain the VPG and APG, calculated by two forward mathematical derivatives, were used to help the PAT extractions. In addition, a 0.5–40 Hz Butterworth bandpass filter and a 0.5–10 Hz Chebyshev II bandpass filter were adopted in the ECG and PPG signal preprocessing, respectively. Note that extreme filtering of ECG and PPG signals can remove the main events, such as the R peaks and systolic waves. However, in this study, filters were selected based on recommendations [23,24,25] from previous studies to emphasize the main events and reduce noise, without causing a phase shift. All filters used in this analysis were zero phase filters to avoid the time shift problem. Detecting the main events correctly plays a major role in examining the PAT effectiveness.

The important characteristic point definition [17,25,26,27] is shown in Figure 3. Note that the MIMIC recordings used in this study were of high quality, where the R peaks in ECG signals and the systolic waves in PPG and ABP signals were salient. Therefore, we are reporting the results based on these signals and used them to carefully check if the PAT and PTT durations are correlated with BP or not. Otherwise, adding noisy signals will bias the results, making it impossible to reach a solid conclusion. In addition, some pathologies have not been taken into account. Because PAT/PTT parameter calculation is based on ECG and PPG morphological feature points, there is no evidence as to whether a disease such as arrhythmia affects the feature point extraction. In future research, participants with certain diseases should be included.

Unlike our previous study [28], which did not rely on detecting features as it fed the PPG signal into a deep neural network to detect hypertension, the present study focused on the correlation between blood pressure and PAT features. According to the arterial wave propagation theory, the heart, arm artery, and finger peripheral arteries play different roles in the transmission of blood. Here, ECG, ABP, and PPG signals reflect the different functions of the cardiovascular organ, especially the blood flow information. Therefore, to represent the different transit paths, three PAT features were defined and calculated: from ECG to ABP (PAT_RS*_), from ECG to PPG (PAT_RO_, PAT_R*a*_, PAT_R*w-1*_, PAT_R*b*_, PAT_RS_), and from ABP to PPG (PTT_S*S_). The detailed feature definitions are shown in Table 2.

### 2.3. Correlation

A correlation analysis was conducted, and the Pearson correlation coefficient was calculated. The Pearson correlation coefficient can measure the strength of the linear relationship between two variables. It ranges from −1 to 1. A positive value indicates a positive correlation, which means that two variables increase or decrease together; a negative value indicates a negative correlation, which means that, as one variable increases, the other decreases. The following formula shows the calculation of the Pearson correlation coefficient, which is generally named as *r*:

(1) $$r=\frac{N\sum xy-\sum x\sum y}{\sqrt{\left(N\sum x^{2}+{\left(\sum x\right)}^{2}\right)\left(N\sum y^{2}+{\left(\sum y\right)}^{2}\right)}}$$

where *N* is the number of samples, and *x* and *y* are two independent variables. Note that the linear correlation is the foundation of the BP regression model. A stable correlation relationship study based on physiological data is the first step in establishing a blood pressure model. Regression analysis is the traditional method of predicting the blood pressure value. In the present study, it will be the first selection to predict blood pressure using the PAT/PTT parameter.

## 3. Results

With the development of wearable health devices, cuff-less BP monitoring is attracting considerable attention [29]. Arterial wave propagation theory (PAT-based) is predominant in cuff-less BP measurement. It should be noted that BP formation is extremely complex and is affected by heart function, artery elasticity, and peripheral vascular network status. Most of these factors affect BP, mainly by affecting the transmission of blood. Therefore, it is feasible to study BP through arterial wave propagation theory. Although many researchers share the opinion that pulse transit time (PAT) has a strong correlation with BP, others have found a weak correlation between PTT/PAT and BP. An in-depth study of correlation analysis is needed.

For the purpose of this research, a large dataset with older subjects was collected from the MIMIC waveform database, which is a public and freely available [30] database with a large number of participants and physiological signals. As mentioned in Section A, although a large quantity of records was collected, many were rejected for reasons such as missing signals, abnormal signals, and extremely low-quality signals. Ultimately, 121 subjects’ records were included in this study. A total of eight arterial wave propagation features were extracted, which included five PAT features from ECG R to PPG characteristics, one PAT feature from ECG R to ABP, one PAT feature from ABP to PPG, and one RR interval feature to represent the process of arterial wave propagation. For a comprehensive analysis, three independent correlation analyses were conducted, including subject by subject, collective beats, and one subject–one excellent beat, respectively. The Pearson correlation coefficient was calculated. Table 3 shows the correlation coefficient for each subject between SBP and the arterial wave propagation features.

Table 4 shows the statistics of Pearson correlation coefficient strength for each subject. The ECG and PPG signals of the 11 subjects with a very strong correlation were synchronized, and the 33 subjects with a very weak correlation were not completely synchronized.

To evaluate the BP correlation of many subjects, a correlation analysis based on the collective extracted beats from all the subjects was implemented. In total, 13,311 beats were used to calculate the Pearson correlation coefficient between the PATs and SBP, as shown in Table 5. Additionally, to avoid impacting point recognition and extraction, one excellent beat for each subject was annotated and extracted, which means that the beat segment of ABP, ECG, and PPG waveforms was excellent and that S* and D* in the ABP waveform; R in the ECG waveform; and O, S, *w*, *a*, and *b* in the PPG, VPG, and APG waveforms, were extracted correctly. Therefore, a total of 121 excellent beats were used to analyze the correlation between BP and arterial wave propagation features, as shown in Table 5.

## 4. Discussion

In this study, a correlation analysis was conducted to evaluate arterial wave propagation theory and explore its effectiveness in monitoring BP. Many researchers have carried out cuff-less BP measurement studies based on arterial wave propagation theory [11,31]. However, different dataset types (private, few participants) and different research methods (calibrated subject by subject, calibration-free) make the correlations between BP and PTT/PAT extremely varied. A comprehensive and clear validation of the correlation between BP and PTT/PAT based on more participants and a public database was needed. This study included the records of 121 subjects. Seven PTT/PAT features and one RR interval feature were defined and extracted from ABP, ECG, PPG, VPG, and APG waveforms. These features represent arterial wave propagation processes, such as PAT_RO_, PAT_R*a*_, PAT_R*w*-1_, PAT_R*b*_, PAT_RS_, PAT_RS*_ (pulse transit time from ECG to ABP and PPG), PTT_S*S_ (pulse transit time from ABP to PPG), and RRI (one heartbeat interval). They were extracted beat by beat for each subject, and the features were then generated.

For different impact analysis, three independent correlation analyses were implemented: subject by subject, whole extracted beats from all subjects, and one excellent beat for each subject. Because of the impact of different heights, weights, genders, ages, and cardiac outputs, etc., the subject-by-subject correlation analysis was conducted first. Table 3 shows the correlation coefficient of each subject. For some subjects, PAT_RS*_ and PAT_R*b*_ showed better correlation strengths, and some subjects showed a weak correlation. Furthermore, different subjects showed significantly different correlations, for example, the correlation coefficient between SBP and PAT_RS*_ for record “12531” was −0.92, and for record “46138” it was just −0.07. A total of 28 subjects had strong correlation strengths, 27 subjects had moderate correlation strengths, and 66 subjects had weak correlation strengths, as shown in Table 4. From these results, it may be seen that the subjects differed in Pearson correlations between the SBP and PAT features. The impacting factors were various, for example, age, gender, height, weight, and cardiovascular disease. Seeberg et al. [32], Li et al. [33], and Martin et al. [3] reported similar results for healthy subjects when they did subject-by-subject correlations. Some subjects in the present study had extremely strong correlations between SBP and PAT, but other subjects showed weak correlations. Therefore, the PAT method for monitoring BP has some limitations for some subjects. Finally, a collective correlation analysis was conducted for all subjects. A total of 13,311 PAT features were extracted from 121 subjects. The Pearson correlation coefficients of SBP, MAP, and DBP, respectively, were calculated, as shown in Table 5.

As Table 5 shows, PAT_R*b*_ yielded a better result, and the other correlation coefficients with SBP, MAP, and DBP were −0.50, −0.42, and −0.31. DBP, respectively, and did not have a good correlation relationship with the PAT features. Therefore, PAT-based features are not a good choice for DBP monitoring or prediction [33]. Although PAT_R*b*_ showed a moderate correlation relationship, it was just −0.50, which is not sufficient to accurately predict BP based on PAT features alone. To validate the PAT-method features effectively, the annotation of signal quality was implemented, and one excellent beat from each subject was used to extract this study’s features.

The Pearson correlation was conducted with these excellent features, as shown in Table 5. As may be seen from Table 5, most of the correlation coefficients were improved. The PAT_R*b*_ was also the best PAT feature, as the collective correlation analysis and its correlation coefficient with SBP, MAP, and DBP were −0.54, −0.42, and −0.28, respectively. The correlation strength was acceptable, but still not very high. Therefore, a dataset with a different group of subjects (healthy or young) and fewer participants could show different correlation strengths, and, as found in the literature, it could be higher (up to −0.75) than in this study, which was based on more and older participants.

Previously, many researchers have defined several PAT/PAT features based on arterial wave propagation theory, which have generally included PAT_RO_, PAT_R*w-1*_, and PAT_RS_. PAT_Rw-1_ has mostly been reported as having the best correlation with BP. In the present study, in line with arterial wave propagation theory, more PAT/PAT features based on ECG, ABP, and PPG waveforms were defined. The paths of the heart, arm artery, and finger micro artery affect BP formation. Therefore, PAT_RS*_ based on ECG and ABP waveforms; PAT_RO_, PAT_R*a*_, PAT_R*w-1*_, PAT_R*b*_, and PAT_RS_ based on ECG and PPG waveforms; and PTT_S*S_ based on ABP and PPG waveforms, can reflect the different transit processes and thereby aid understanding of the arterial wave propagation theory. As may be seen from Table 3, PAT_RS*_ has a stronger correlation relationship than other features for most subjects. This indicates that the relationship between BP and PAT is more stable in the aorta artery than in the peripheral artery. Peripheral artery diseases are common in the elderly population, which affects the arterial wave propagation and makes the correlation relationship weak.

It should be noted that, for some subjects, the finger PPG-based features are easily distorted in the distal region. Even more serious is that blunt valleys and peaks make O and S difficult to detect clearly, which causes a significantly lower correlation strength with BP than others. The *a*, *w*, and *b* have steep and stable peaks and valleys that help to calculate PAT correctly. Table 5 shows that PAT_R*a*_, PAT_R*w-1*_, and PAT_R*b*_ are obviously higher than PAT_RO_ and PAT_RS_, which is similar to the conclusions of many previous studies [34]. Therefore, for correct PAT extraction from a larger dataset of older participants, PAT_R*b*_, PAT_R*w-1*_, or PAT_R*a*_ are better choices.

Table 6 presents a review of the literature on the Pearson correlation relationship between BP and PAT features, using the MIMIC database. It is clear that studies disagree about the strength of the correlation. These researchers may not have used objective measures to select recordings from the MIMIC database, which led to their reporting strong correlations between PAT and BP.

The best way to create a database is to ensure that all the biosignals have been simultaneously collected and synchronized. However, if there is a need to use the MIMIC database for evaluating BP, we suggest extracting asynchronicity-dependent features, such as PPG features or ECG features. One other recommendation is to apply a synchrony measure, as carried out by Martínez et al. [38] on ECG, ABP, and PPG signals and select the recordings that achieve the highest synchronicity. As mentioned before, the focus of this paper is to examine the PAT duration for the existing MIMIC database because there are many papers that used the database as is for assessing blood pressure. One of the next steps is to investigate the synchronicity over the asynchronous MIMIC database; however, it is not an easy problem to address.

## 5. Conclusions

Pulse arrival time (PAT) is a potential parameter in continuous BP monitoring. The ability to track BP on a beat to beat basis and analyze its patterns may have novel clinical applications. However, there are some limitations if the MIMIC database is used for its evaluation. Interestingly, PAT_R*b*_ showed a better correlation with BP than PAT_RO_ (the traditional measure). It was also found that the correlation between SBP and PAT was better than between DBP and PAT. As in the existing literature, the overall analysis showed that there is a negative correlation between BP and PAT features. However, the MIMIC database is not recommended for examining any synchronicity-dependent features, such as the PAT interval, for predicting blood pressure, without the prior step of a synchronicity check.

## Figures and Tables

**Table 1:** Excluded and included MIMIC records in the study. Note that ABP is the arterial blood pressure, ECG is the electrocardiogram, and PPG is the photoplethysmogram signal.

Type	Record Description
**Missing signal** **(Excluded records)**	**Without ABP waveform (**no abp signal or just a straight line in whole record, 284 records**):** a44002, a44005, a44007, a44011, a44026, a44027, a44032, a44044, a44053, a44059, a44060, a44061, a44073, a44080, a44083, a44084, a44094, a44096, a44101, a44107, a44109, a44110, a44116, a44127, a44129, a44132, a44134, a44136, a44141, a44145, a44159, a44170, a44178, a44180, a44182, a44188, a44192, a44197, a44200, a44204, a44209, a44213, a44220, a44222, a44226, a44241, a44256, a44291, a44294, a44304, a44306, a44307, a44314, a44322, a44337, a44358, a44382, a44383, a44391, a44402, a44405, a44406, a44428, a44459, a44463, a44466, a44491, a44507, a44512, a44518, a44537, a44539, a44559, a44561, a44570, a44571, a44578, a44582, a44602, a44610, a44611, a44612, a44617, a44625, a44639, a44648, a44651, a44659, a44674, a44710, a44712, a44718, a44727, a44740, a44745, a44755, a44757, a44762, a44772, a44791, a44807, a44813, a44830, a44838, a44863, a44868, a44895, a44907, a44911, a44921, a44941, a44946, a44947, a44979, a45014, a45050, a45062, a45065, a45071, a45072, a45077, a45091, a45095, a45097, a45105, a45121, a45133, a45158, a45161, a45177, a45184, a45228, a45229, a45260, a45262, a45264, a45265, a45275, a45276, a45298, a45301, a45303, a45346, a45355, a45369, a45375, a45379, a45427, a45464, a45515, a45516, a45524, a45532, a45538, a45541, a45546, a45554, a45562, a45582, a45583, a45601, a45617, a45619, a45629, a45639, a45663, a45665, a45676, a46002, a46008, a46009, a46013, a46017, a46074, a46104, a46147, a46154, a46165, a46200, a46208, a46236, a46280, a46292, a46302, a46313, a46329, a46339, a46365, a46383, a46387, a46391, a46430, a46433, n10036, n10309, s00618, s00631, s01049, s01158, s01621, s01791, s01978, s02586, s02906, s02981, s03345, s03386, s03695, s03920, s04641, s04833, s04904, s04906, s05030, s05345, s05742, s06116, s06381, s08061, s08122, s08396, s08915, s09473, s09798, s09870, s09920, s09993, s10152, s10475, s10667, s10799, s11004, s11342, s11388, s12508, s12589, s12632, s13599, s14058, s14325, s14579, s14714, s14936, s15298, s15852, s16112, s16139, s16391, s17112, s17394, s17497, s17875, s18082, s18123, s18225, s18393, s18727, s18975, s19093, s19726, s19827, s20612, s23038, s23762, s23824, s25954, s26039, s26211, s26330, s26712, s27060, s27194, s27212, s27338, s27551, s27638, s27689, s28083, s28611, s28808, s28863, s28927, s29057, s29093. **Without ECG waveform (**no ecg signal or just a straight line in whole record, 9 records**):** a44243, a44264, a44558, a44903, a45005, a45046, a45468, a46377, n10139. **Without PPG waveform (**no ppg signal or just a straight line in whole record, 22 records**):** a44087, a44162, a44166, a44190, a44238, a44385, a44469, a44588, a44716, a45159, a46269, s00652, s04324, s06158, s06539, s07445, s09058, s10842, s14266, s20196, s23238, s28625.
**Abnormal signal** **(Excluded records)**	**Abnormal ABP waveform (**114 records**):** a44046, a44227, a44331, a44486, a44591, a44599, a44694, a44859, a44891, a44900, a45013, a45357, a45401, a45461, a45467, a45493, a45519, a45535, a46108, a46133, a46192, a46379, a46423, s01840, s01855, s01949, s06946, s07251, s07614, s07654, s08142, s10049, s12351, s14533, s14947, s15545, s21247, s22585, s24942, s25411, s26709, s26978, s27084, s27193, s27232, s27890, s28075, s28702, s28897, s29199 s1004, s2063, s2614, s2858, s3617, s3744, s4331, s4802, s6875, s9258, s10629, s11431, s12878, s15488, s17421, s17582, s18274, s21002, s21202, s22364, s22462, s23363, s23876, s25284, s25724, s27192, s27425, s27585, s27687, s27696, s28044, s28048, s28364, s28774, s29215, a44033, a44047, a44089, a44106, a44113, a44117, a44139, a44164, a44215, a44318, a44332, a44348, a44349, a44368, a44378, a44442, a44452, a44505, a44585, a44644, a44992, a45045, a45222, a45495, a45511, a45648, a46098, a46176, a46289 **Abnormal ECG waveform (**21 records**):** s2703, s7415, s8281, s17795, s22418, s27542, s27636, s28079, s28189, s28354, s28507, s28698, s28707, s28762, s28901, s28905, a44082, a44398, a44474, a44715, a45060 **abnormal PPG waveform (**7 records**):** s15480, s27374, a44041, a44167, a44228, a44426, a44508
**Good quality signals** **(Included records)**	s10464, s11187, s11727, s12174, s12531, s13600, s01501, s15218, s15716, s15902, s01606, s16129, s17848, s18642, s18970, s19578, s19700, s20726, s02104, s21730, s22335, s23201, s02458, s02513, s26897, s27241, s27337, s27434, s27436, s27446, s27648, s27833, s27845, s27887, s28077, s28187, s28499, s28510, s28758, s28775, s28813, s28882, s28910, s29102, s29120, s29127, s29167, s03039, a44088, a44104, a44118, a44165, a44171, a44173, a44201, a44223, a44233, a44347, a44409, a44422, a44432, a44458, 44496, a44526, a44572, a44590, a44598, a44601, a44615, a44616, a44623, a44626, a44629, a44640, a44647, a44671, a44704, a44758, a44763, a44810, a44839, a44902, a44981, a45049, a45098, a45140, a45181, a45186, a45212, a45227, a45311, a45343, a45353, a45384, a45426, a45456, a45487, a45533, a45550, a45572, a45627, a45636, a45641, a45645, a46122, a46138, a46216, a46230, a46297, a46303, a46416, a46424, s04679, s06581, s06692, s07614, s00801, s08141, s08318, s09124, s00946

**Table 2:** Artery wave propagation feature definitions.

Name	Start Point	End Point	Description
**SBP**	-	-	Systolic blood pressure
**MAP**	-	-	Mean arterial pressure
**DBP**	-	-	Diastolic blood pressure
**PAT_RO_**	ECG R	PPG O	Pulse transit time from R wave to O wave
**PAT_R*a*_**	ECG R	APG *a*	Pulse transit time from R wave to *a* wave
**PAT_R*w*-1_**	ECG R	PPG *w*_-1_	Pulse transit time from R wave to *w_-1_* wave
**PAT_R*b*_**	ECG R	APG *b*	Pulse transit time from R wave to *b* wave
**PAT_RS_**	ECG R	PPG S	Pulse transit time from R wave to S wave
PAT_RS*_	ECG R	ABP S*	Pulse transit time from R wave to S* wave
PTT_S*S_	ABP S*	PPG S	Pulse transit time from S* wave to S wave
**RRI**	ECG R	ECG R	R-R interval

**Table 3:** The Pearson correlation coefficient for each subject between blood pressure and arterial wave propagation features. Note: The “subject ID” is the same as in the MIMIC database. “#PATs” means the quantity of extracted beat records from each subject. Note, SBP refers to systolic blood pressure, PAT refers to pulse arrival time, PTT refers to pulse transient time, RRI refers to R-R interval extracted from ECG signal, STD refers to standard deviation, and *r* refers to Pearson’s correlation coefficient.

Index	Subject ID	# PATs	*r* (SBP, PAT_RO_)	*r* (SBP, PAT_R*a*_)	*r* (SBP, PAT_R*w*-1_)	*r* (SBP, PAT_R*b*_)	*r* (SBP, PAT_RS_)	*r* (SBP, PAT_RS*_)	*r* (SBP, PTT_S*S_)	*r* (SBP, RRI)
**1**	801	149	0.01	−0.02	−0.05	−0.05	−0.18	−0.37	−0.10	−0.36
**2**	946	77	−0.39	−0.57	−0.42	−0.12	−0.14	−0.46	0.16	0.04
**3**	1501	120	−0.35	−0.31	−0.46	−0.25	0.13	−0.72	0.36	−0.03
**4**	1606	55	−0.22	−0.45	−0.37	−0.38	−0.16	−0.36	0.03	−0.05
**5**	2104	95	0.03	−0.07	−0.09	−0.18	−0.11	−0.28	0.04	0.05
**6**	2458	84	−0.04	−0.25	−0.07	0.00	0.37	0.08	0.30	0.50
**7**	2513	79	−0.20	−0.57	−0.52	−0.52	−0.54	−0.70	−0.21	−0.14
**8**	3039	53	−0.45	−0.55	−0.50	−0.43	−0.37	−0.75	0.40	−0.13
**9**	4679	140	−0.43	−0.53	−0.62	−0.46	−0.63	−0.66	−0.45	−0.54
**10**	6581	52	0.03	−0.10	−0.15	−0.10	−0.09	0.08	−0.11	0.64
**11**	6692	154	−0.75	−0.73	−0.74	−0.68	−0.68	−0.53	−0.53	0.16
**12**	7614	62	−0.02	−0.24	0.02	−0.07	0.10	−0.33	0.29	−0.01
**…**	…	…	…	…	…	…	…	…	…	…
**…**	…	…	…	…	…	…	…	…	…	…
**110**	45627	77	−0.27	−0.05	0.00	−0.09	0.30	0.36	0.19	−0.10
**111**	45636	53	−0.35	−0.41	−0.29	−0.29	−0.29	−0.09	−0.28	−0.09
**112**	45641	117	−0.45	−0.40	−0.43	−0.22	−0.44	−0.21	−0.32	−0.02
**113**	45645	118	−0.27	−0.36	−0.43	−0.51	−0.49	0.08	−0.63	−0.06
**114**	46122	94	0.01	−0.62	−0.60	−0.44	−0.27	−0.42	−0.11	0.50
**115**	46138	119	−0.30	−0.44	−0.48	−0.52	0.31	−0.07	0.41	0.13
**116**	46216	35	0.02	0.28	0.31	0.28	0.32	0.25	0.20	0.28
**117**	46230	73	−0.60	−0.72	−0.76	−0.78	−0.75	−0.92	0.27	0.35
**118**	46297	170	−0.50	−0.47	−0.43	−0.44	−0.40	−0.85	0.05	−0.17
**119**	46303	83	−0.37	−0.34	−0.30	−0.33	0.05	−0.27	0.17	−0.08
**120**	46416	88	−0.44	−0.27	0.05	0.08	0.05	−0.40	0.15	0.53
**121**	46424	122	−0.16	−0.16	−0.30	−0.19	−0.21	−0.54	0.15	−0.04
	Mean ± STD	110 ± 45	−0.20 ± 0.25	−0.30 ± 0.25	−0.30 ± 0.27	−0.26 ± 0.25	−0.12 ± 0.31	−0.30 ± 0.36	0.04 ± 0.27	−0.03 ± 0.31

**Table 4:** Summary of Pearson correlation coefficient (*r*) strengths. Note: This statistics table is calculated based on the PAT_RS*_ value, which scored the highest overall correlation with systolic blood pressure.

Strength of Correlation	Range of Absolute Correlation Coefficient (*r*)	Count of Subjects
Very strong	0.8–1.0	11
Strong	0.6–0.79	17
Moderate	0.4–0.59	27
Weak	0.2–0.39	33
Very weak	0–0.19	33

**Table 5:** The Pearson correlation coefficient between blood pressure and arterial wave propagation features, using total extracted beats of all subjects and one excellent beat from each subject. Note, SBP refers to systolic blood pressure, DBP refers to diastolic blood pressure, MAP refers to mean arterial pressure, PAT refers to pulse arrival time, PTT refers to pulse transient time, RRI refers to R-R interval extracted from ECG signal, and *r* refers to Pearson’s correlation coefficient.

Trial	BP	# PATs	*r* (SBP, PAT_RO_)	*r* (SBP, PAT_R*a*_)	*r* (SBP, PAT_R*w*-1_)	*r* (SBP, PAT_R*b*_)	*r* (SBP, PAT_RS_)	*r* (SBP, PAT_RS*_)	*r* (SBP, PTT_S*S_)	*r* (SBP, RRI)
**Collective beats all subjects**	**SBP**	13311	−0.41	−0.47	−0.49	−0.50	−0.43	−0.41	−0.22	−0.08
	**MAP**	13311	−0.30	−0.38	−0.40	−0.42	−0.35	−0.29	−0.23	−0.02
	**DBP**	13311	−0.20	−0.27	−0.29	−0.31	−0.26	−0.17	−0.20	0.03
**One excellent beat per subject**	**SBP**	121	−0.50	−0.51	−0.52	−0.54	−0.46	−0.39	−0.28	0.01
	**MAP**	121	−0.37	−0.38	−0.41	−0.42	−0.37	−0.28	−0.26	0.01
	**DBP**	121	−0.23	−0.23	−0.27	−0.28	−0.25	−0.16	−0.20	0.01

**Table 6:** Studies on the Pearson’s correlation relationship between blood pressure and pulse arrival time features, using the MIMIC database. Note, SBP refers to systolic blood pressure, DBP refers to diastolic blood pressure, MAP refers to mean arterial pressure, PAT refers to pulse arrival time, *r* refers to Pearson’s correlation coefficient, and N/R refers to not reported.

Author (s)	Number of Subjects	Analysis	Relationship	Pearson Correlation Coefficient (*r*)
**This study, 2019**	121	Collective	(SBP, PAT_R*b*_) (MAP, PAT_R*b*_) (DBP, PAT_R*b*_)	−0.54 −0.42 −0.28
**Kachuee et al. [35], 2017**	942	Collective	(BP, PAT)	N/R
**Yoon et al. [36], 2017**	23	Subject by subject	(SBP, PAT_Rw-1_) (MAP, PAT_Rw-1_) (DBP, PAT_Rw-1_)	−0.53 ± 0.32 −0.49 ± 0.34 −0.40 ± 0.35
**He et al. [37], 2014**	100	Collective	(SBP, PAT_RO_) (MAP, PAT_RO_) (DBP, PAT_RO_)	−0.7 N/R N/R
**Choi et al. [19], 2013**	25	Collective	(SBP, PAT_RS_) (DBP, PAT_RS_)	−0.71 −0.69
